# Retraction: Dexmedetomidine postconditioning suppresses myocardial ischemia/reperfusion injury by activating the SIRT1/mTOR axis

**DOI:** 10.1042/BSR-2019-4030_RET

**Published:** 2022-09-14

**Keywords:** Dexmedetomidine, EX527, mTOR, Myocardial ischemia/reperfusion, SIRT1

This Retraction follows an Expression of Concern relating to this article previously published by Portland Press. This article is being retracted from *Bioscience Reports* at the request of the authors following receipt of a notification from a reader, alerting the Editorial Office to two regions of the figure 6b MI/R + Dex + EX527 panel that also appear in another paper by different authors (“Protective effect of tanshinone IIA against cardiac hypertrophy in spontaneously hypertensive rats through inhibiting the Cys-C/Wnt signaling pathway” (Feng et al 2017) doi: 10.18632/oncotarget.14328). The Editorial Office also has concerns regarding the western blot images. The authors wish to retract the article, and the Editor-in-Chief and Editorial Board agree with the retraction.

